# Perception of Neighborhood Safety and Maternal and Neonatal Health Outcomes

**DOI:** 10.1001/jamanetworkopen.2023.17153

**Published:** 2023-05-31

**Authors:** Julia G. Carter, Joe M. Feinglass, Lynn M. Yee

**Affiliations:** 1 Department of Obstetrics and Gynecology, Division of Maternal-Fetal Medicine, Northwestern University Feinberg School of Medicine, Chicago, Illinois; 2 Department of Medicine, Division of General Internal Medicine and Geriatrics, Northwestern University Feinberg School of Medicine, Chicago, Illinois

## Abstract

This cross-sectional study examines whether perceived neighborhood safety is independently associated with adverse birth outcomes.

## Introduction

Neighborhood-level exposures have found to be associated with reduced birth weight, preterm birth, small-for-gestational-age neonates, perinatal depression, and inadequate prenatal care.^1^ Poor neighborhood safety perception has been found to be associated with increased blood pressure, higher levels of stress, decreased participation in physical activity, smoking, and consuming alcohol. This cross-sectional study aimed to determine whether perceived neighborhood safety is independently associated with adverse birth outcomes.

## Methods

The Pregnancy Risk Assessment Monitoring System conducts surveys 2 to 4 months after delivery with linked birth certificate data.^2^ In 2016 to 2020, a question related to perceived neighborhood safety was used for respondents in Illinois, Louisiana, Minnesota, Missouri, Pennsylvania, Rhode Island, Virginia, and Wisconsin. Respondents were asked, “During the 12 months before your new baby was born, how often did you feel unsafe in the neighborhood where you lived?” Answers were categorized as always or often unsafe, sometimes unsafe, rarely unsafe, and never unsafe. Data were weighted to reflect live, singleton birth populations of 8 states. Pregnancy Risk Assessment Monitoring System data are deidentified and publicly available and institutional review board exempt; thus, informed consent was not sought, in accordance with 45 CFR §46. This study follows the STROBE reporting guidelines for cross-sectional studies.

Birth outcomes analyzed included low birth weight (LBW; <2500 g), self-reported depressive symptoms during pregnancy or post partum, attending more than 8 prenatal care visits, attending a postpartum visit, and breastfeeding for 8 weeks or more. Multivariable logistic and Poisson regression models were used to test the independent significance of perceived neighborhood safety for the likelihood of outcomes after controlling for maternal age, race and ethnicity, marital status, educational attainment, prepregnancy insurance status, body mass index, prepregnancy smoking, preexisting gestational hypertension or diabetes, history of depression, and interpersonal physical and emotional abuse. An item, “In the 12 months before you got pregnant with your new baby, did any of the following people push, hit, slap, kick, choke, or physically hurt you in any other way?” was used to assess interpersonal physical and emotional abuse.

## Results

After 1829 exclusions, 29 987 respondents were included, reflecting a weighted 8-state population of 2 172 915; 16.3% of respondents were non-Hispanic Black, 12.5% were Hispanic, 63.4% reported more than 12 years of education, 37.3% reported annual household income higher than $60 000, 23.7% had births covered by Medicaid, 35.5% reported prepregnancy abuse, 14.2% had a history of depression, 9.3% had gestational diabetes, 12.9% had hypertensive disorders of pregnancy, 18.7% reported prepregnancy smoking, and 27.3% had obesity.

The majority of respondents (78.0%) reported they never felt unsafe, 13.7% responded rarely feeling unsafe, 5.3% sometimes felt unsafe, and 3.0% reported they always or often felt unsafe. A total of 6.5% of respondents had LBW neonates, 21% reported perinatal depressive symptoms, 85.9% attended more than 8 prenatal care visits, 89.5% attended a postpartum visit, and 53.9% reported breastfeeding for 8 weeks or more. Compared with respondents who never felt unsafe, those reporting always or often feeling unsafe had 23% higher odds of having a LBW neonate, 100% higher odds of perinatal depressive symptoms, and 10% lower odds of attending more than 8 prenatal care visits with no significant association with attending a postpartum visit or breastfeeding (Table).

**Table.** Association of Perceived Neighborhood Safety and Maternal and Neonatal Health Outcomes From Pregnancy Risk Assessment Monitoring System From 8 States, 2016-2020^a^^,^^b^

Perceived neighborhood safety	Low birth weight, aIRR (95% CI)^c^	Perinatal depression, aOR (95% CI)^d^	aIRR (95% CI)		
			Prenatal care >8 visits	Postpartum visit	Breastfeeding ≥8 wk
Always or often unsafe	1.23 (1.02-1.50)	2.00 (1.55-2.58)	0.90 (0.85-0.94)	0.96 (0.92-1.00)	1.00 (0.90-1.12)
Sometimes unsafe	1.03 (0.87-1.20)	1.42 (1.15-1.76)	0.95 (0.91-0.98)	0.98 (0.96-1.01)	1.09 (1.01-1.16)
Rarely unsafe	0.90 (0.79-1.01)	1.18 (1.01-1.37)	0.98 (0.96-1.00)	1.00 (0.98-1.01)	1.10 (1.06-1.14)
Never unsafe	1 [Reference]	1 [Reference]	1 [Reference]	1 [Reference]	1 [Reference]

Abbreviations: aIRR, adjusted incidence rate ratio; aOR, adjusted odds ratio.

^a^ Perceived neighborhood safety was determined by the response to the Pregnancy Risk Assessment Monitoring System (PRAMS) Phase 8 2016-2020 survey question from 8 states (Illinois, Louisiana, Minnesota, Missouri, Pennsylvania, Rhode Island, Virginia, and Wisconsin), “During the 12 months before your new baby was born, how often did you feel unsafe in the neighborhood where you lived?”

^b^ Covariates included maternal age, race and ethnicity, income, education level, prepregnancy insurance status, prepregnancy smoking, preexisting hypertension, preexisting diabetes, preexisting depression, and interpersonal physical and emotional abuse.

^c^ Low birth weight was defined as a birth weight of less than 2500 g.

^d^ Perinatal depression was defined as answering either often or always to either of the following questions either during pregnancy or postpartum: “How often have you felt down, depressed or hopeless?” and “How often have you had little interest or little pleasure in doing things you usually enjoyed?”

## Discussion

Although this cross-sectional study could not assess causation, the association of poorer perceived neighborhood safety with less prenatal care utilization is supported by a previous study from Canada.^3^ Patients from communities with high levels of neighborhood violence can benefit from universal trauma-informed care, assistance with resource needs, and help in scheduling and attending appointments.^4^

These findings have policy implications. Social and economic interventions designed to combat neighborhood and domestic violence may be more beneficial in reducing adverse pregnancy outcomes than biomedical interventions.^5^ There are increasing calls to reduce expensive and often counterproductive police crime prevention and mass incarceration strategies.^6^ To more effectively support the health of pregnant people and their families, we need to invest in strengthening low-income communities, including resources for community-based violence prevention and mental health support.

## Notes

Supplement.
Data Sharing Statement
