# Supplementary material for: Perception of Neighborhood Safety and Maternal and Neonatal Health Outcomes
Source: JAMA Netw Open. 2023 May 31;6(5):e2317153. doi: 10.1001/jamanetworkopen.2023.17153 (PMC10233411; doi:10.1001/jamanetworkopen.2023.17153)
Supplement: Supplement. — Data Sharing Statement [file jamanetwopen-e2317153-s001.pdf]

Data Sharing Statement

Carter. Perception of Neighborhood Safety and Maternal and Neonatal Health Outcomes.
JAMA Netw Open. Published May 31, 2023. doi:10.1001/jamanetworkopen.2023.17153

Data
Data available: Yes
Data types: Deidentified participant data
How to access data: Data can be shared upon reasonable request.
When available: With publication

Supporting Documents
Document types: None

Additional Information
Who can access the data: N/A
Types of analyses: N/A
Mechanisms of data availability: N/A
Any additional restrictions: N/A
